# Body mass index associated with childhood and adolescent high‐risk B‐cell acute lymphoblastic leukemia risk: A Children’s Oncology Group report

**DOI:** 10.1002/cam4.3334

**Published:** 2020-07-24

**Authors:** Taumoha Ghosh, Michaela Richardson, Peter M. Gordon, Justin R. Ryder, Logan G. Spector, Lucie M. Turcotte

**Affiliations:** ^1^ Division of Epidemiology and Clinical Research Department of Pediatrics University of Minnesota Minneapolis MN USA; ^2^ Division of Hematology/Oncology Department of Pediatrics University of Minnesota Minneapolis MN USA; ^3^ Center for Pediatric Obesity Medicine University of Minnesota Minneapolis MN USA

**Keywords:** childhood ALL, obesity, risk factors

## Abstract

**Background:**

Obesity is a risk factor for many adulthood cancers, but its role in childhood, adolescent, and young adult (AYA) cancer is unknown. Childhood and AYA acute lymphoblastic leukemia (ALL) incidence and obesity prevalence have shown concurrent increases. We sought to identify whether obesity may be a risk factor for childhood and AYA ALL.

**Methods:**

Characteristics from individuals with ALL, aged 2‐30 years, diagnosed 2004‐2017 and treated on Children's Oncology Group (COG) protocols with available pre‐treatment anthropometric data (N = 4726) were compared to National Health and Nutrition Examination Survey controls (COG AALL17D2). Body mass index (BMI) was defined using standard CDC definitions. Multivariate conditional logistic regression assessed associations between BMI and ALL with additional analyses stratified by sex and race/ethnicity.

**Results:**

Among cases (72% high‐risk (HR) B‐ALL, 28% T‐ALL), 5% had underweight, 58% normal weight, 17% overweight, and 20% obesity. Underweight (OR 2.11, 95% CI 1.56‐2.85) and obesity (OR 1.32, 95% CI 1.15‐1.53) were associated with B‐ALL diagnosis. Specifically, obesity was associated with B‐ALL among males (OR 1.57, 95% CI 1.30‐1.91) and Hispanic children (OR 1.78, 95% CI 1.39‐2.29). Obesity was also associated with central nervous system (CNS) involvement.

**Conclusion:**

Pre‐treatment obesity is associated with HR B‐ALL among males and Hispanics, as well as with CNS involvement, suggesting common physiology between obesity and leukemogenesis. An association between underweight and ALL was confirmed, likely due to cancer‐associated wasting. These results have important public health implications for obesity prevention and treatment in children and adolescents to reduce cancer risk.

## Graphical abstract

Deviations in body mass index (underweight and obesity) at the time of diagnosis were significantly associated with a diagnosis of pediatric high‐risk (HR) B‐cell acute lymphoblastic leukemia (B‐ALL) compared to normal weight. Specifically, obesity was associated with HR B‐ALL in males and Hispanics. Obesity was also significantly associated with ALL central nervous system (CNS) involvement.

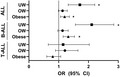

## 1 INTRODUCTION

Acute lymphoblastic leukemia (ALL) is the most common form of malignancy among children, adolescents, and young adults (AYA).^1^ Since the 1970s, the incidence of ALL has steadily increased without a clear etiology.^1^ Aside from inherited syndromes and certain perinatal factors including cesarean delivery, advanced maternal age, and higher birthweight,^2^, ^3^, ^4^ few endogenous or environmental risk factors have been identified that explain the temporal changes in the incidence of ALL among this age group.^5^

Over the same time frame that childhood and AYA ALL has increased, a concurrent rise in the prevalence of obesity has been observed.^6^ There is growing evidence in adulthood cancers associating multiple host characteristics and lifestyle exposures with cancer risk.^7^ Obesity has been strongly linked to 13 adulthood cancers^8^ and is potentially associated with others, including hematologic malignancies.^9^, ^10^, ^11^ Obesity now accounts for up to 20% of all adulthood cancers^12^ and obesity‐associated cancers are increasingly being diagnosed in younger adult populations.^13^, ^14^ Despite this, associations between obesity and childhood cancer risk have not been well studied. Recent studies of children and AYA with obesity and ALL have focused on disease outcomes rather than etiology, and have shown increased risk for end of induction residual disease and relapse.^15^ Given the associations between adult‐onset cancers and obesity, the inferior ALL outcomes in children and AYA with obesity, and the parallel increases in ALL incidence and obesity prevalence in children and AYA, we hypothesized that pre‐treatment obesity in children and AYA is associated with the development of high‐risk (HR) ALL and that it is associated with HR ALL features, such as higher initial white blood cell count, CNS disease, and prognostic cytogenetic characteristics.

## 2 MATERIALS AND METHODS

### 2.1 Study population

**FIGURE 1:**
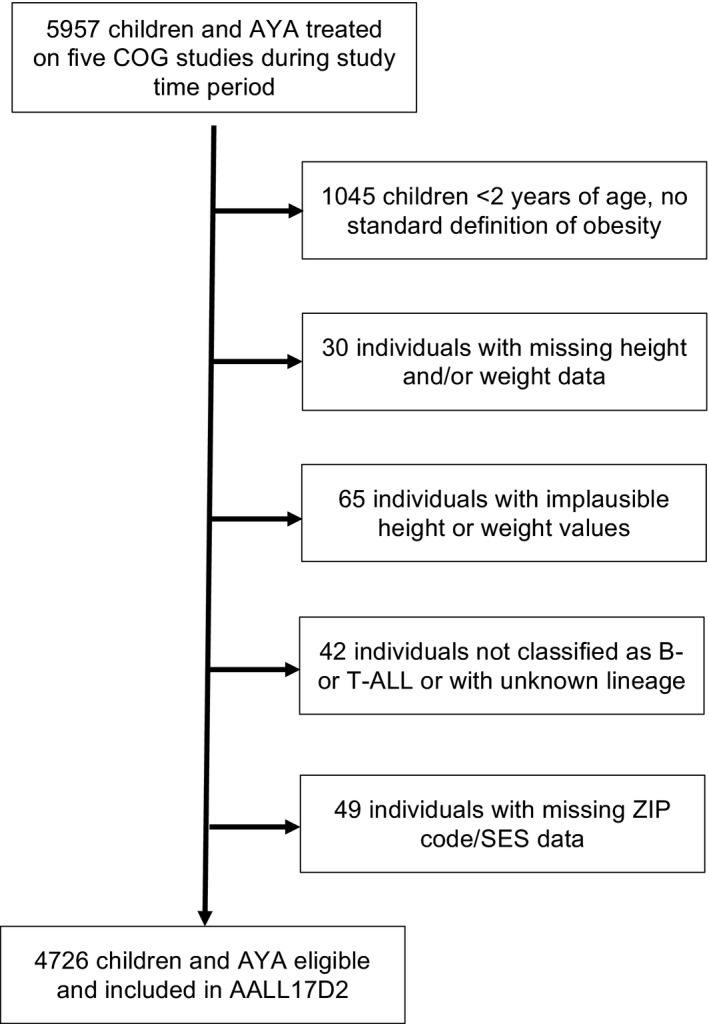
CONSORT diagram depicting exclusion of cases due to i) specific criteria of age < 2 y due to no standard definition of obesity, ii) missing anthropometric data, iii) implausible anthropometric data, iv) lack of B‐ALL or T‐ALL classification, or unknown lineage, v) and missing ZIP code/SES data. ALL, acute lymphoblastic leukemia; AYA, adolescent and young adult; COG, Children's Oncology Group; SES, socioeconomic status

Children and AYA aged 2‐30 years with newly diagnosed B‐cell precursor or T‐cell ALL (B‐ALL or T‐ALL) enrolled on the five Children's Oncology Group (COG) frontline treatment studies that collected pre‐treatment height and weight were included in the analysis (COG ALL17D2, Figure 1, Table S1). Height and weight at diagnosis were not available for AALL0932, the recently completed large standard‐risk (SR) B‐ALL protocol.

The study population distribution by COG treatment protocol included: 2516 participants from AALL0232 (53.2%), 1322 participants from AALL0434 (28.0%), 29 participants from AALL0622 (0.6%), 153 participants from AALL07P4 (3.2%), and 706 participants from AALL1131 (14.9%). Controls were selected from the National Health and Nutrition Examination Survey (NHANES),^16^ a nationally representative sample of US adults and children. Controls were matched 1:1 to ALL cases by age (within 1 year), sex, and race/ethnicity, given these biologic factors are associated with both obesity and leukemia risk. Year of sampling/diagnosis (within 10 years) was also matched to control for secular trends in obesity prevalence. All data were de‐identified.

### 2.2 Variables of interest

Anthropometric data for cases and controls were used to calculate body mass index (BMI; kg/m^2^). For cases, these measures were taken at the time of diagnosis prior to receiving treatment. For individuals < 20 years of age, BMI‐percentiles were determined based on sex and age using the Center for Disease Control and Prevention (CDC) standards, and for individuals ≥ 20 years of age calculated BMI was used. Pediatric (2‐<20 years of age) and adult (20‐30 years of age) definitions were used to classify individuals as underweight, normal weight, overweight, and obesity (Table 1A).

**TABLE 1:** Definitions of variables of interest. (A) BMI categories; (B) SES; (C) Leukemia characteristics

(A)		
	Pediatric (<20 y of age) (based on BMI for sex and age)	Adult (≥20 y of age) (BMI, kg/m^2^)
Underweight	<5th percentile	<18.5
Normal Weight	5th‐<85th percentile	18.5‐24.9
Overweight	85th‐<95th percentile	25.0‐29.9
Obesity	>95th percentile	>30.0

(B)		
	Cases	Controls
Low SES	>20% of the population in a ZIP code living at or below the federal poverty threshold	Poverty Income Ratio < 1.00
High SES	<20% of the population in a ZIP code living at or below the federal poverty threshold	Poverty Income Ratio > 1.00

(C)
ALL lineage (B‐ or T‐cell)
CNS status at diagnosis (CNS 1, 2, or 3)
CNS 1: Absence of leukemic blasts in CSF on cytospin preparation, regardless of WBC number
CNS 2: Less than 5/uL WBCs in CSF and cytospin‐positive for blasts (or ≥5/uL WBCs but negative by Steinherz/Bleyer algorithm)
CNS 3: At least 5/uL WBCs in CSF and cytospin‐positive for blasts and/or clinical signs of CNS leukemia
WBC count at diagnosis (<50 or >50 × 10^9^/L)
Hypodiploidy (<44 chromosomes, DNA index <0.81)
*BCR‐ABL1* fusion
*ETV6‐RUNX1* translocation
*KMT2A (MLL)* rearrangement
Double Trisomy (Trisomies of chromosomes 4 and 10)

Abbreviations: ALL, acute lymphoblastic leukemia; BMI, body mass index; CNS, central nervous system; CSF, cerebrospinal fluid; SES, socioeconomic status; WBC, white blood cell.

Demographic data including age (month and year of birth), sex, race/ethnicity, and socioeconomic status (SES) (Table 1B) were collected for cases and controls. ZIP code analysis for SES was used for cases, based on available data in case files. Additional SES data were not available through the COG. Poverty Income Ratio (PIR) was collected by NHANES and is used by the US Census to evaluate families’ income status compared to the poverty threshold. PIR was used as the measure of SES for controls. Leukemia characteristics were collected for ALL cases, including lineage (B‐ or T‐cell), centrally‐reviewed cytogenetics, DNA ploidy, white blood cell (WBC) count at diagnosis, and central nervous system (CNS) status at diagnosis (Table 1C).

### 2.3 Statistical analysis

Demographic characteristics as well as distribution of BMI categories were compared between controls (NHANES) and ALL cases (COG) using chi‐square testing or Fisher's exact test. Univariate analyses were performed to examine associations between BMI and ALL characteristics. Multivariable conditional logistic regression models, adjusting for SES, sampling year, and age at sampling/diagnosis were used to calculate odds ratios (OR) to examine associations between obesity and specific ALL disease characteristics with additional analyses stratified by sex and race/ethnicity. Statistical analyses were performed using SAS, version 9.4. For all analyses, *P* < .05 was considered statistically significant.

## 3 RESULTS

Among 4726 eligible cases (Figure 1), 38% were female, median age at diagnosis was 11.3 years (range 2.0‐30.0 years), 72% had B‐cell lineage disease, and low SES was present in 14%. Underweight was observed in 5% of cases, 58% were normal weight, 17% were overweight, and 20% were obese (Table 2). Difference in sampling year distribution among cases and controls (*P* < .0001) as well as difference in age groups between cases and controls (*P* = .001) were noted despite matching due to a limited number of available controls and differing enrollment periods of the COG studies, though the absolute differences between cases and controls for these factors were small. Cases were more likely to be classified as having high SES than controls (*P* < .0001) and BMI category distributions differed between cases and controls (*P* < .0001).

**TABLE 2:** Descriptive characteristics of the study population

Factor	COG cases N = 4726 (%)	NHANES controls N = 4726 (%)	Χ^2^ *P*‐value
Sex			—
Male	2909 (61.6)	2909 (61.6)	
Female	1817 (38.5)	1817 (38.5)	
Race/Ethnicity			—
Non‐Hispanic, white	2750 (58.2)	2750 (58.2)	
Non‐Hispanic, black	419 (8.9)	419 (8.9)	
Hispanic	1140 (24.1)	1140 (24.1)	
Multiple/Other/Unknown	417 (8.8)	417 (8.8)	
Sampling year			<.0001
2003‐2004	170 (3.6)	351 (7.4)	
2005‐2006	774 (16.4)	633 (13.4)	
2007‐2008	1046 (22.1)	785 (16.6)	
2009‐2010	1255 (26.6)	850 (18.0)	
2011‐2012	554 (11.7)	705 (14.9)	
2013‐2014	435 (9.2)	731 (15.5)	
2015‐2016	492 (10.4)	671 (14.2)	
Age (mo)			.006*
Mean (SD)	135.7 (64.8)	139.3 (65.7)	
Range	24.0‐360.0	24.0‐360.0	
Age (y)			.001
Children (2‐<10)	1753 (37.1)	1665 (35.2)	
Adolescent (10‐<20)	2794 (59.1)	2813 (59.5)	
Young adult (20‐30)	179 (3.8)	248 (5.3)	
Therapeutic study			
AALL0232 (B‐cell)	2516 (53.2)	—	
AALL0434 (T‐cell)	1322 (28.0)	—	
AALL07P4 (B‐cell)	153 (3.2)	—	
AALL0622 (B‐cell)	29 (0.6)	—	
AALL1131 (B‐cell)	706 (14.9)	—	
SES status^a^			<.0001
Low	655 (13.9)	1309 (27.7)	
High	4071 (86.1)	3417 (72.3)	
BMI category^b^, ^*^			<.0001
Underweight	242 (5.1)	148 (3.1)	
Normal weight	2752 (58.2)	2891 (61.2)	
Overweight	784 (16.6)	758 (16.0)	
Obesity	948 (20.1)	929 (19.7)	

Abbreviations: AYA, adolescent and young adult; BMI, body mass index; COG, Children's Oncology Group; NHANES, National Health and Nutrition Examination Survey; SD, standard deviation; SES, socioeconomic status.

^a^ SES defined as follows: For controls—Low SES was defined as PIR ≤ 1.00; High SES was defined as PIR > 1.00; For cases—Low SES was defined as ≥ 20% of the population in a zip code living at or below the federal poverty threshold High SES was defined as < 20% of the population in a zip code living at or below the federal poverty threshold

^b^ BMI categorized as follows: For subjects < 20 y old: Underweight: <5th BMI percentile; Normal weight: 5th to < 85th BMI percentile; Overweight: ≥85th to < 95th BMI percentile; Obesity: ≥95th BMI percentile.For subjects ≥ 20 y old: Underweight: <18.5 kg/m^2^; Normal weight: 18.5‐<25 kg/m^2^; Overweight: ≥25 ‐ <30 kg/m^2^; Obesity: ≥30 kg/m^2^.

* *P*‐value from *t* Test

Associations between prognostic ALL characteristics and BMI categories were assessed among cases (Table 3). CNS status was associated with BMI category (*P* = .01), with patients with obesity experiencing a higher proportion of CNS 2 and 3 status compared to other BMI categories. BMI category was also associated with ALL lineage (*P* = .006), as well as the presence of trisomies of chromosomes 4 and 10 (*P* < .0001). Other prognostic characteristics were not statistically associated with BMI category.

**TABLE 3:** ALL prognostic characteristics by BMI category for all cases

Factor	Underweight N = 242	Normal Weight N = 2752	Overweight N = 784	Obesity N = 948	*Χ* ^2^ *P*‐value^b^, *
CNS status					.02
1	201 (83.1)	2234 (81.2)	613 (78.2)	729 (76.9)	
2	27 (11.2)	407 (14.8)	136 (17.4)	164 (17.3)	
3	12 (5.0)	105 (3.8)	33 (4.2)	52 (5.5)	
Unknown	2 (0.8)	6 (0.2)	2 (0.3)	3 (0.3)	
Immunophenotype					.003
B‐Precursor	179 (74.0)	1929 (70.1)	574 (73.2)	721 (76.1)	
T‐Cell	63 (26.0)	823 (29.9)	210 (26.8)	227 (24.0)	
Hypodiploid					.58
Yes	4 (1.7)	67 (2.4)	21 (2.7)	29 (3.1)	
No	236 (97.5)	2673 (97.1)	757 (96.6)	915 (96.5)	
Unknown	2 (0.8)	11 (0.4)	5 (0.6)	3 (0.3)	
Indeterminate	‐	1 (0.04)	1 (0.1)	1 (0.1)	
BCR_ABL1					.09
Yes	16 (6.6)	105 (3.8)	30 (3.8)	29 (3.1)	
No	205 (84.7)	2419 (87.9)	681 (86.9)	824 (86.9)	
Unknown	21 (8.7)	228 (8.3)	73 (9.3)	95 (10.0)	
ETV6_RUNX1					.35
Yes	16 (6.6)	219 (8.0)	56 (7.1)	60 (6.3)	
No	161 (66.5)	1877 (68.2)	549 (70.0)	667 (70.4)	
Unknown	65 (26.9)	656 (23.8)	179 (22.8)	221 (23.3)	
MLL					.10
Yes	2 (0.8)	84 (3.1)	22 (2.8)	36 (3.8)	
No	208 (86.0)	2319 (84.3)	648 (82.7)	775 (81.8)	
Unknown	32 (13.2)	349 (12.7)	114 (14.5)	137 (14.5)	
Double trisomy					<.0001
Yes	43 (17.8)	270 (9.8)	54 (6.9)	69 (7.3)	
No	162 (66.9)	2118 (77.0)	616 (78.6)	732 (77.2)	
Unknown	37 (15.3)	364 (13.2)	114 (14.5)	147 (15.5)	
WBC					.21
<50	135 (56.0)	1548 (56.3)	454 (58.1)	569 (60.0)	
≥50	106 (44.0)	1202 (43.7)	327 (41.9)	378 (39.9)	

Abbreviations: ALL, acute lymphoblastic leukemia; BMI, body mass index; CNS, central nervous status; WBC, white blood cell count.

* Unknown and Indeterminate categories not included in *Χ* ^2^ test.

**FIGURE 2:**
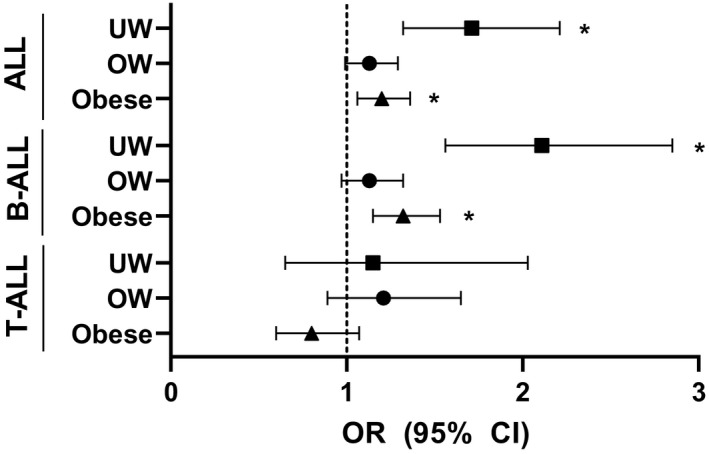
Odds of ALL by BMI Category. Calculated by multivariable conditional logistic regression. Model adjusted for: SES (High, Low), sampling year and age at sampling/diagnosis. Normal weight used as reference. Asterisks (*) indicates significant findings. Abbreviations: ALL, Acute Lymphoblastic Leukemia; OW, Overweight; UW, Underweight

Multivariable conditional logistic regression was performed to investigate the association between BMI category and ALL diagnosis, using normal weight as the reference group and adjusting for SES, sampling year, and age at sampling/diagnosis. Underweight at diagnosis was associated with ALL (B‐ and T‐cell) diagnosis (OR 1.71, 95% CI 1.3 −2.21). An association was also identified between obesity and ALL (B‐ and T‐cell) diagnosis (OR 1.20, 95% CI 1.06‐1.36; Figure 2). When B‐ALL and T‐ALL were considered separately, BMI category was only associated with B‐ALL (underweight, OR 2.11, 95% CI 1.56‐2.85 and obesity, OR 1.32, 95% CI 1.15‐1.53; Figure 2). As such, further analyses were focused on B‐ALL.

**FIGURE 3:**
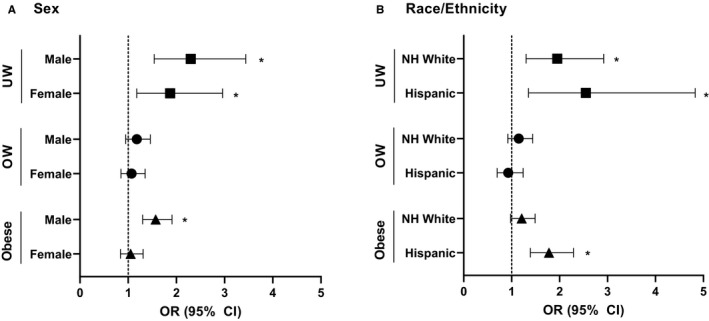
A, Odds of B‐ALL by BMI category by sex. Calculated by multivariable conditional logistic regression. Model adjusted for: SES (High, Low), sampling year and age at sampling/diagnosis. Normal weight used as reference. Asterisks (*) indicates significant findings. Abbreviations: ALL, Acute Lymphoblastic Leukemia; OW, Overweight; UW, Underweight. B, Odds of B‐ALL by BMI category by race/ethnicity. Calculated by multivariable conditional logistic regression. Model adjusted for: SES (High, Low), sampling year and age at sampling/diagnosis. Normal weight used as reference. Asterisks (*) indicates significant findings. Abbreviations: ALL, Acute Lymphoblastic Leukemia; NH, Non‐Hispanic; OW, Overweight; UW, Underweight

Subsequent multivariable conditional logistic regression analyses stratified by sex with sex‐specific controls, identified associations between B‐ALL diagnosis among males (Figure 3A) with underweight (OR 2.30, 95% CI 1.54‐3.44) and obesity (OR 1.57, 95% CI 1.30‐1.91). Stratification by age group (2 to <10, 10 to <20, and 20‐30 years; Figure S1) showed an association between males with obesity and B‐ALL among the 2 to <10 years age group (OR 1.61, 95% CI 1.12‐2.31) and 10 to <20 years age group (OR 1.47, 95% CI 1.16‐1.87). An association was also seen between males with underweight status and ALL among the 10 to <20 years age group (OR 2.48, 95% CI 1.49 ‐ 4.13). Associations were not identified for females or for males aged 20‐30 years.

Additional multivariable conditional logistic regression analyses were stratified by race/ethnicity (Figure 3B). Analyses could not be stratified for Non‐Hispanic (NH) Black given limited numbers. A significant association was identified between B‐ALL diagnosis and NH Whites with underweight status (OR 1.95, 95% CI 1.30‐2.92), but not with overweight or obesity. Among Hispanic ethnicity, B‐ALL diagnosis was associated with obesity (OR 1.78, 95% CI 1.39‐2.29) and underweight (OR 2.55, 95% CI 1.35‐4.83). When further stratified by age group (Figure S2), associations with B‐ALL diagnosis were seen in those aged 10 to <20 years for NH White with underweight (OR 2.19, 95% CI 1.27) and for Hispanics with underweight (OR 2.19, 95% CI 1.01‐4.73) and Hispanics with obesity (OR 1.74, 95% CI 1.29‐2.34). No significant associations were seen in other age groups when analyses were stratified by race/ethnicity.

**FIGURE 4:**
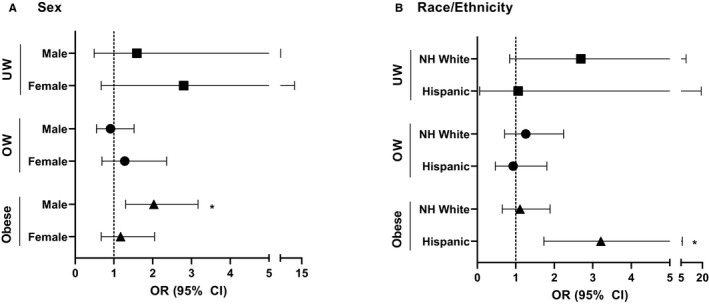
A, Odds of B‐ALL CNS Involvement by BMI category and sex. Calculated by multivariable conditional logistic regression. Model adjusted for: SES (High, Low), sampling year and age at sampling/diagnosis. Normal weight used as reference. Asterisks (*) indicates significant findings. Abbreviations: ALL, Acute Lymphoblastic Leukemia; OW, Overweight; UW, Underweight. B, Odds of B‐ALL CNS Involvement by BMI category and race/ethnicity. Calculated by multivariable conditional logistic regression. Model adjusted for: SES (High, Low), sampling year and age at sampling/diagnosis. Normal weight used as reference. Asterisks (*) indicates significant findings. Abbreviations: ALL, Acute Lymphoblastic Leukemia; NH, Non‐Hispanic; OW, Overweight; UW, Underweight

Further multivariable analyses assessed associations between BMI category and B‐ALL characteristics, again stratified by sex and race/ethnicity. Males with obesity had a stronger association with moderate to high levels of CNS involvement (CNS 2‐3) at diagnosis (OR 2.03, 95% CI 1.30‐3.17) compared to no CNS involvement (CNS 1) (OR 1.49, 95% CI 1.20‐1.85) (Figure 4A). These associations were not seen in females. Among Hispanic ethnicity (Figure 4B), obesity was more strongly associated with CNS 2‐3 (OR 3.21, 95% CI 1.73‐5.95) compared to CNS 1 (OR 1.60, 95% CI 1.21‐2.11). These associations were not seen in NH White. Associations between other prognostic B‐ALL characteristics and BMI category were assessed and are available in Tables S2 and S3.

## 4 DISCUSSION

Here we demonstrate the novel findings that pre‐treatment obesity among children is associated with HR B‐ALL diagnosis, specifically among males and Hispanics, particularly within the adolescent age group, and that obesity is also associated with risk for ALL CNS involvement. This study also confirms the previously identified association between underweight status and ALL,^17^ which is felt to be secondary to detrimental physiologic effects of untreated ALL (ie weight loss prior to diagnosis resulting in underweight status at diagnosis) and may result in an underestimation of BMI status prior to the development of ALL.

The association between pre‐treatment obesity and HR B‐ALL diagnosis suggests potential common physiology in both obesity and leukemogenesis. Inflammation has been theorized to play a role in linking obesity and oncogenesis across a variety of cancers as it alters adipokine release, cytokine expression, insulin resistance, and results in other adverse metabolic adaptations.^18^, ^19^, ^20^, ^21^, ^22^ For leukemia, multiple epidemiologic studies have demonstrated an association between obesity and development of leukemia in adults.^23^ The biologic relationship between obesity and leukemogenesis is likely multifactorial. Diet‐induced obesity accelerated ALL progression in murine models, which showed accompanying elevated serum levels of insulin, leptin and IL‐6.^24^ Insulin is a potent growth factor that can increase ALL cell proliferation in vivo,^25^ and adults with diabetes are at increased risk for many cancers, including leukemia.^26^, ^27^ Given the comorbidity of diabetes and obesity, hyperinsulinemia and insulin resistance may therefore be important mediators of leukemogenesis in the setting of obesity. Additionally, leptin is a pro‐inflammatory, pro‐angiogenic, and pro‐tumorigenic adipokine, which has been shown to stimulate multiple leukemia cell types.^18^, ^28^

Obesity‐associated inflammation results in tissue remodeling and increases free fatty acids leading to increased oxidative stress and recruitment of pro‐inflammatory macrophages and upregulation of inflammatory cytokines IL‐1, IL‐6, and TNF‐α.^29^, ^30^ IL‐6 plays a role in B cell proliferation and differentiation, which may impact B‐lineage leukemogenesis.^24^ Obesity‐associated changes in the inflammatory microenvironment may lead to molecular and genetic alterations, resulting in leukemia development or progression. Inflammation is of interest for leukemia etiology, as well as therapy, particularly given the increasing use of immunotherapies for B‐ALL treatment. An important opportunity for future study will be assessing the effect of increasing BMI or severe obesity on leukemogenesis; unfortunately, we were not sufficiently powered to evaluate this in the present study.

The male sex‐specific association between obesity and B‐ALL also suggests a role for sex hormones in leukemogenesis. Males have a higher leukemia incidence than females,^1^ which was also seen in our study population. The association seen in this study was present among pre‐adolescents and adolescents (10 to <20 years). Adult studies have demonstrated a link between estrogen and cancer, and adipose tissue can increase circulating levels of estrogen by conversion of androgens.^18^, ^20^ Sex hormones also regulate cellular differentiation, proliferation, and apoptosis.^18^, ^20^ The identified association between obesity and B‐ALL diagnosis among pre‐pubertal and pubertal males may be a result of excess of sex hormones as they enter puberty resulting in higher levels of estrogen leading to leukemogenesis. While this suggests females should be at higher risk for leukemia, it is possible females have a greater tolerance for circulating estrogen given higher baseline levels compared to males. Additionally, a recent study of metabolomic profiles in youth with obesity demonstrated a unique profile among post‐pubertal males, suggesting an additional avenue for further study.^31^ The identified association between obesity and B‐ALL diagnosis in pubertal and post‐pubertal males may indicate leukemia in adolescents is more biologically similar to that seen in adults compared to children.

This study did not include SR B‐ALL cases, which include the majority of pre‐pubertal children (<10 years of age); thus, the opportunity to assess associations between obesity and leukemia in pre‐pubertal children is limited. It is also critical to point out that BMI does not measure body fatness and is a poor proxy for body fatness unless children and adolescents have severe obesity.^32^ Although males and females have similar body fat until puberty onset with similar increases during the entire pubertal window, later changes result in females having greater fat mass as adults.^33^ Higher fat deposition in female adults may explain why some adult studies have found obesity is associated with leukemia diagnosis in both sexes.^23^

Obesity was associated with B‐ALL diagnosis in Hispanic children and adolescents in our study. Obesity rates are highest among Hispanic children and they are at higher risk for obesity‐related complications.^16^, ^34^, ^35^, ^36^ Over the past two decades ALL rates have increased in Hispanic children at a greater rate than NH children,^37^, ^38^, ^39^ particularly for adolescents aged 15 to 19 years,^37^ which is the age group in which our study identified an association of B‐ALL diagnosis and obesity among Hispanic youth. Different ethnic groups have different percent body fat with similar BMIs.^40^, ^41^ Hispanics, particularly Mexican Americans, have higher body fat percentage compared to NH White and NH Black counterparts, which may contribute to oncogenesis.^41^ Given similar temporal trends in obesity rates and ALL incidence in Hispanic youth and higher rates of adiposity and worse outcomes for ALL among Hispanic children, it is possible there is an underlying biologic mechanism placing Hispanic children at risk for B‐ALL in the setting of obesity.

Pre‐treatment obesity was associated with CNS B‐ALL involvement at diagnosis. Several prior studies have linked obesity with inferior outcomes in childhood ALL.^15^, ^42^, ^43^ It is possible that the CNS, in the setting of obesity may provide a niche for ALL cells to escape immune surveillance and chemotherapy. Moderate CNS involvement with ALL (CNS 2) in the setting of obesity may be due to technical difficulty during a diagnostic lumbar puncture (ie obtaining peripheral blood elements in the sample), the biologic gradient seen in our study (though not statistically significant due to the limited number of CNS positive cases) suggests this finding is real and not purely due to technique. Additionally, other studies have shown obesity can impact neuro‐inflammation and the permeability of the blood brain barrier^44^, ^45^, ^46^, ^47^ suggesting a biological mechanism for ALL penetration into the CNS in patients who are obese.

Important limitations of this study must be considered. The COG studies with anthropometric data were only for HR ALL, resulting in an unavoidable sampling bias and limiting the generalizability of our findings to SR B‐ALL, which is the most common type of ALL seen in young children. Additionally, sampling years for the COG ALL studies was not perfectly matched to those of controls, which is important given the secular trends of obesity, although these trends often span decades. Similarly, the controls had a larger proportion of individuals with low SES. This may be secondary to selection bias that is inherent in the NHANES participant selection; however, prior studies using both the Surveillance, Epidemiology, and End Results program and Minnesota Cancer Surveillance System have demonstrated positive, although smaller, associations between higher SES and ALL diagnosis.^48^, ^49^ Notably, different methods were used for assessing SES in cases (ZIP code) and controls (PIR), which may have resulted in slightly different SES distributions in the two groups. Additionally, we were unable to compare clinical characteristics, including BMI, of COG clinical trial participants and non‐participants; however, nearly 70% of children and adolescents diagnosed with ALL participate in a COG clinical trial.^50^

Additional limitations include limited availability of cytogenetic data for this analysis, precluding assessments for associations between Philadelphia‐like cytogenetics and obesity. Many of the cytogenetic abnormalities considered in this study are more common in SR B‐ALL, which was not evaluated in this study. Similarly, there were a limited number of T‐ALL cases, so it is possible an association between T‐ALL and obesity at diagnosis was not detected due to lack of power. There were limited participants ≥ 20 years of age, limiting adequate assessment of the association between obesity and ALL among young adults. Lastly, we were unable to assess or control for underlying germline mutations or inherited predisposition syndromes, which can impact both leukemogenesis and obesity risk. Despite these limitations, a positive association between HR B‐ALL and obesity was found.

In conclusion, the present study demonstrated a novel association between obesity at diagnosis and new diagnosis of HR B‐ALL, particularly in males and Hispanics. It suggests opportunities for future research to determine whether other pediatric malignancies are also associated with obesity. It also could have important public health implications for obesity prevention and treatment efforts in children and adolescents to reduce future cancer risk. Additionally, this study has implications for the CNS microenvironment in pediatric patients with obesity and ALL. Further studies addressing sex hormones and pro‐inflammatory cytokines and their roles in leukemogenesis are needed, as are studies to assess underlying genetic changes that may contribute to obesity and leukemogenesis among Hispanic children. Additional mechanistic studies could lead to novel therapeutic strategies in the future that would benefit children with obesity and HR B‐ALL.

Funding Contributions: This work was supported by the University of Minnesota Department of Pediatrics Vikings Award and by a grant from the National Cancer Institute (K08CA234232 [LMT]).

## CONFLICT OF INTEREST

The authors of this manuscript certify that they have NO affiliations with or involvement in any organization or entity with any financial interest (such as honoraria; educational grants; participation in speakers’ bureaus; membership, employment, consultancies, stock ownership, or other equity interest; and expert testimony or patent‐licensing arrangements), or non‐financial interest (such as personal or professional relationships, affiliations, knowledge or beliefs) in the subject matter or materials discussed in this manuscript.

## AUTHOR CONTRIBUTIONS

All persons who meet authorship criteria are listed as authors, and all authors certify that they have participated sufficiently in the work to take public responsibility for the content, including participation in the concept, design, analysis, writing, or revision of the manuscript.

## ETHICAL REVIEW STATEMENT

Ethical approval was not sought from our institutional review board or ethics committee prior to commencing this study.

## Supporting information

Fig S1

Fig S2

Table S1

Table S2

Table S3

## Data Availability

Data sharing is not applicable to this article as no new data were created or analyzed in this study.
