# Supplementary figures and images for: Body mass index associated with childhood and adolescent high‐risk B‐cell acute lymphoblastic leukemia risk: A Children’s Oncology Group report
Source: Cancer Med. 2020 Jul 24;9(18):6825–35. doi: 10.1002/cam4.3334 (PMC7520304; doi:10.1002/cam4.3334)

## A. Male

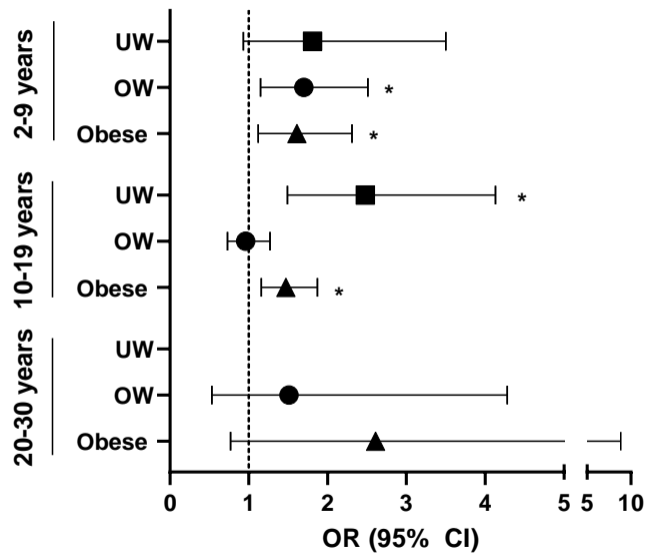

## B. Female

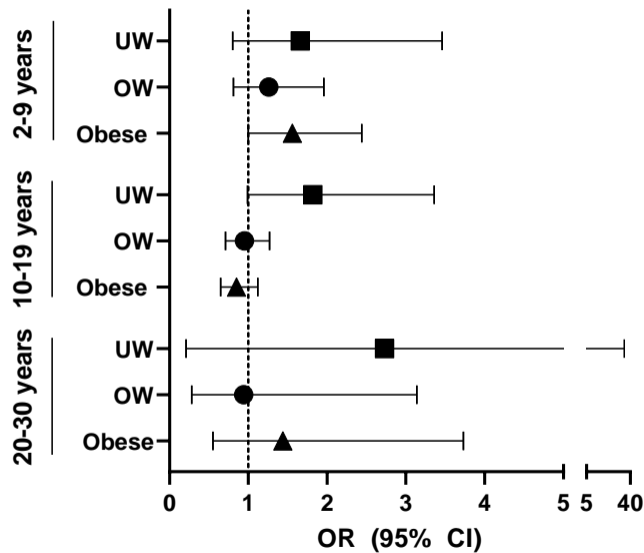

Supplement: Supplementary file 1 — Fig S1 [file CAM4-9-6825-s001.pdf]

## A. NH White

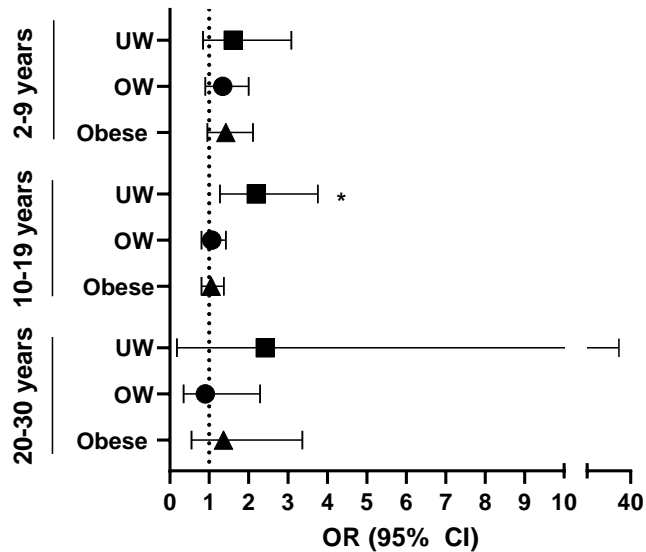

## B. Hispanic

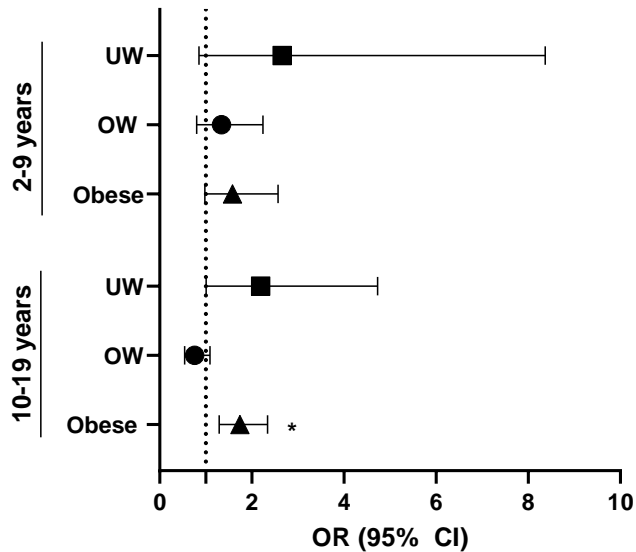

Supplement: Supplementary file 2 — Fig S2 [file CAM4-9-6825-s002.pdf]
