# Supplementary material for: Body mass index associated with childhood and adolescent high‐risk B‐cell acute lymphoblastic leukemia risk: A Children’s Oncology Group report
Source: Cancer Med. 2020 Jul 24;9(18):6825–35. doi: 10.1002/cam4.3334 (PMC7520304; doi:10.1002/cam4.3334)
Supplement: Supplementary file 3 — Table S1 [file CAM4-9-6825-s003.docx]

Supplementary Table 1. Children’s Oncology Group (COG) ALL treatment studies with available pre-treatment height and weight data.

| **COG Protocol** | **COG Study Title** | **Years Study Open to Enrollment** |
| --- | --- | --- |
| AALL0232 | High Risk B-precursor Acute Lymphoblastic Leukemia (ALL) | 2003-2011 |
| AALL0434 | Intensified Methotrexate, Nelarabine (Compound 506U78; IND#52611) and Augmented BFM Therapy for Children and Young Adults with Newly Diagnosed T-cell Acute Lymphoblastic Leukemia (ALL) or T-cell Lymphoblastic Lymphoma | 2007-2014 |
| AALL0622 | Intensified Tyrosine Kinase Inhibitor Therapy (Dasatinib: IND# 73969, NSC# 732517) in Philadelphia Chromosome Positive Acute Lymphoblastic Leukemia (ALL) | 2008-2012 |
| AALL07P4 | A Pilot Study of Intravenous EZN-2285 (SC-PEG E. coli L-asparaginase, IND# 100594) or Intravenous Oncaspar® in the Treatment of Patients with High-Risk Acute Lymphoblastic Leukemia (ALL) | 2008-2012 |
| AALL1131 | A Phase III Randomized Trial for Newly Diagnosed High Risk B-Lymphoblastic Leukemia (B-ALL) Including a Stratum Evaluating Dasatinib (IND#73789, NSC#732517) in Patients with Ph-like Tyrosine Kinase Inhibitor (TKI) Sensitive Mutations | 2012-2019 |
