# Supplementary material for: Body mass index associated with childhood and adolescent high‐risk B‐cell acute lymphoblastic leukemia risk: A Children’s Oncology Group report
Source: Cancer Med. 2020 Jul 24;9(18):6825–35. doi: 10.1002/cam4.3334 (PMC7520304; doi:10.1002/cam4.3334)
Supplement: Supplementary file 4 — Table S2 [file CAM4-9-6825-s004.docx]

Supplementary Table 2: Odds of B-ALL Prognostic Characteristics by BMI category and sex

|  | | **Male** | | **Female** | |
| --- | --- | --- | --- | --- | --- |
|  |  | **OR (95% CI)** | **p-value** | **OR (95% CI)** | **p-value** |
| **Hypodiploid** | **Underweight** | 1.79 (0.15-21.38) | 0.65 | NE | NE |
|  | **Normal weight** | Ref |  | Ref |  |
|  | **Overweight** | 0.64 (0.18-2.23) | 0.48 | 3.71 (0.79-17.43) | 0.10 |
|  | **Obese** | 1.50 (0.51-4.45) | 0.47 | 0.70 (0.18-2.7) | 0.60 |
| **BCR-ABL1** | **Underweight** | 8.17 (0.70-94.94) | 0.09 | 1.87 (0.18-19.95) | 0.60 |
|  | **Normal weight** | Ref |  | Ref |  |
|  | **Overweight** | 0.80 (0.26-2.45) | 0.70 | 2.38 (0.64-8.82) | 1.20 |
|  | **Obese** | 0.73 (0.3-1.76) | 0.48 | 1.35 (0.44-4.17) | 0.60 |
| **ETV6-RUNX1** | **Underweight** | 2.06 (0.56-7.55) | 0.27 | 1.31 (0.32-5.31) | 0.70 |
|  | **Normal weight** | Ref |  | Ref |  |
|  | **Overweight** | 1.41 (0.67-2.99) | 0.37 | 0.68 (0.30-1.54) | 0.36 |
|  | **Obese** | 1.02 (0.53-1.98) | 0.95 | 1.44 (0.69-2.98) | 0.33 |
| **MLL** | **Underweight** | 0.57 (0.03-12.03) | 0.72 | NE |  |
|  | **Normal weight** | Ref |  | Ref |  |
|  | **Overweight** | 1.09 (0.19-6.23) | 0.92 | 0.32 (0.06-1.88) | 0.21 |
|  | **Obese** | 3.22 (0.52-19.93) | 0.21 | 3.96 (0.50-31.72) | 0.19 |
| **Double Trisomy (chromosomes 4 & 10)** | **Underweight** | **11.19 (3.23-38.74)** | **0.0001** | 2.27 (0.65-7.95) | 0.20 |
|  | **Normal weight** | Ref |  | Ref |  |
|  | **Overweight** | 0.71 (0.32-1.58) | 0.41 | 1.19 (0.58-2.41) | 0.64 |
|  | **Obese** | 0.92 (0.47-1.8) | 0.82 | 0.90 (0.44-1.81) | 0.76 |
| **WBC > 50K** | **Underweight** | **2.31 (1.20-4.45)** | **0.0123** | 1.87 (0.93-3.76) | 0.08 |
|  | **Normal weight** | Ref |  | Ref |  |
|  | **Overweight** | 1.39 (0.97-1.99) | 0.07 | 1.02 (0.68-1.52) | 0.93 |
|  | **Obese** | **1.71 (1.23-2.38)** | **0.0016** | 1.25 (0.85-1.84) | 0.25 |

Abbreviations: CI, Confidence Interval; NE, Not Evaluable; NH, Non-Hispanic; OR, Odds Ratio; Ref, Reference; WBC, White Blood Cell Count
