# Supplementary material for: Body mass index associated with childhood and adolescent high‐risk B‐cell acute lymphoblastic leukemia risk: A Children’s Oncology Group report
Source: Cancer Med. 2020 Jul 24;9(18):6825–35. doi: 10.1002/cam4.3334 (PMC7520304; doi:10.1002/cam4.3334)
Supplement: Supplementary file 5 — Table S3 [file CAM4-9-6825-s005.docx]

Supplementary Table 3: Odds of B-ALL Prognostic Characteristics by BMI category and race/ethnicity

|  | | **NH White** | | **Hispanic** | |
| --- | --- | --- | --- | --- | --- |
|  |  | **OR (95% CI)** | **p-value** | **OR (95% CI)** | **p-value** |
| **Hypodiploid** | **Underweight** | 2.58 (0.04-174.9) | 0.66 | NE | NE |
|  | **Normal weight** | Ref |  | Ref |  |
|  | **Overweight** | 1.43 (0.42-4.83) | 0.57 | 0.83 (0.15-4.63) | 0.83 |
|  | **Obese** | 1.57 (0.43-5.71) | 0.49 | 0.89 (0.20-3.98) | 0.88 |
| **BCR-ABL1** | **Underweight** | 2.58 (0.39-17.21) | 0.33 | NE |  |
|  | **Normal weight** | Ref |  | Ref |  |
|  | **Overweight** | 1.18 (0.39-3.52) | 0.77 | 1.08 (0.18-6.60) | 0.93 |
|  | **Obese** | 0.66 (0.26-1.67) | 0.38 | 3.47 (0.50-NE) | 0.21 |
| **ETV6-RUNX1** | **Underweight** | 0.97 (0.28-3.42) | 0.97 | 0.60 (0.05-7.37) | 0.69 |
|  | **Normal weight** | Ref |  | Ref |  |
|  | **Overweight** | 0.64 (0.31-1.29) | 0.21 | 1.14 (0.31-4.11) | 0.85 |
|  | **Obese** | 1.08 (0.57-2.07) | 0.81 | 1.67 (0.66-4.22) | 0.28 |
| **MLL** | **Underweight** | 0.48 (0.04-6.61) | 0.59 | NE |  |
|  | **Normal weight** | Ref |  | Ref |  |
|  | **Overweight** | 0.36 (0.07-1.77) | 0.21 | NE | 0.50 |
|  | **Obese** | 2.07 (0.38-11.19) | 0.40 | 5.51 (0-NE) | 0.79 |
| **Double Trisomy (chromosomes 4 & 10)** | **Underweight** | **7.26 (2.32-22.7)** | **0.0007** | 3.65 (0.81-NE) | 0.09 |
|  | **Normal weight** | Ref |  | Ref |  |
|  | **Overweight** | 0.95 (0.47-1.93) | 0.90 | 0.75 (0.29-1.96) | 0.56 |
|  | **Obese** | 1.20 (0.63-2.28) | 0.58 | 0.91 (0.38-2.19) | 0.83 |
| **WBC > 50K** | **Underweight** | **2.32 (1.22-4.42)** | **0.0103** | 2.01 (0.71-5.73) | 0.19 |
|  | **Normal weight** | Ref |  | Ref |  |
|  | **Overweight** | 1.11 (0.77-1.60) | 0.57 | 1.03 (0.63-1.68) | 0.92 |
|  | **Obese** | 1.24 (0.85-1.81) | 0.26 | 1.89 (1.25-2.84) | **0.0023** |

Abbreviations: CI, Confidence Interval; NE, Not Evaluable; NH, Non-Hispanic; OR, Odds Ratio; Ref, Reference; WBC, White Blood Cell Count
